# Predictors of Endotoxin Levels in U.S. Housing

**DOI:** 10.1289/ehp.11759

**Published:** 2008-10-16

**Authors:** Peter S. Thorne, Richard D. Cohn, Deepak Mav, Samuel J. Arbes, Darryl C. Zeldin

**Affiliations:** 1 Environmental Health Sciences Research Center, College of Public Health, University of Iowa, Iowa City, Iowa, USA;; 2 Constella Group, LLC, Durham, North Carolina, USA;; 3 Division of Intramural Research, National Institute of Environmental Health Sciences, Research Triangle Park, North Carolina, USA

**Keywords:** allergens, asthma triggers, endotoxin, house dust, housing characteristics, indoor air, lipopolysaccharide, microorganism-associated molecular pattern, predictive model, reservoir dust

## Abstract

**Background:**

The relationship of domestic endotoxin exposure to allergy and asthma has been widely investigated. However, few studies have evaluated predictors of household endotoxin, and none have done so for multiple locations within homes and on a national scale.

**Objectives:**

We assayed 2,552 house dust samples in a nationwide study to understand the predictors of household endotoxin in bedroom floors, family room floors, beds, kitchen floors, and family room sofas.

**Methods:**

Reservoir house dust from five locations within homes was assayed for endotoxin and demographic and housing information was assessed through questionnaire and onsite evaluation of 2,456 residents of 831 homes selected to represent national demographics. We performed repeated-measures analysis of variance (rANOVA) for 37 candidate variables to identify independent predictors of endotoxin. Meteorologic data were obtained for each primary sampling unit and tested as predictors of indoor endotoxin to determine if wetter or warmer microclimates were associated with higher endotoxin levels.

**Results:**

Weighted geometric mean endotoxin concentration ranged from 18.7 to 80.5 endotoxin units (EU)/mg for the five sampling locations, and endotoxin load ranged from 4,160 to 19,500 EU/m^2^. Bivariate analyses and rANOVA demonstrated that major predictors of endotoxin concentration were sampling location in the home, census division, educational attainment, presence of children, current dog ownership, resident-described problems with cockroaches, food debris, cockroach stains, and evidence of smoking observed by field staff. Low household income entered the model if educational attainment was removed.

**Conclusion:**

Increased endotoxin in household reservoir dust is principally associated with poverty, people, pets, household cleanliness, and geography.

Household exposure to endotoxin has emerged as an important factor in the development and severity of nonatopic asthma (Michel et al. 1996; Thorne et al. 2005) while apparently reducing the likelihood of allergic sensitization and lessening the chance of developing eosinhilic asthma (Braun-Fahrländer et al. 2002; Ernst and Cormier 2000; Klintberg et al. 2001). However, there is strong evidence that occupational endotoxin exposure is a potent agent for the development and exacerbation of neutrophilic asthma, asthmalike syndrome, and organic dust toxic syndrome (Thorne and Duchaine 2007).

Endotoxin is an amphiphilic outer-cell-wall component of gram-negative bacteria that is a potent inflammatory agent and asthma trigger. As a microorganism-associated molecular pattern (MAMP), endotoxin is recognized by the innate immune system through an evolutionarily conserved pathway. Endotoxin recognition and signal amplification occur through a series of endotoxin–protein and protein–protein interactions leading to activation of toll-like receptor-4 (TLR4), with resulting inflammation (Sigsgaard et al. 2008). Key molecules for the endotoxin recognition pathway include lipopolysaccharide-binding protein, CD14, and MD-2 (Hađina et al. 2008). A number of polymorphisms have been identified that affect expression of key molecules in the inflammatory cascade and that may play a role in responsiveness to endotoxin. Thus, dose, coexposures to other MAMPs and allergens, and genetic susceptibility may be important predictors of response to indoor endotoxin.

Because of the importance of limiting endotoxin exposures, particularly among asthmatic individuals, several studies have evaluated the predictors of endotoxin concentration in house dust or endotoxin loading of surfaces in homes (Bischof et al. 2002; Gehring et al. 2004; Park et al. 2001; Wickens et al. 2003). In general, these studies have been confined to a particular geographic area, demographic group, or type of housing, and most have been limited to either the family room floor dust or bedding. Because of the targeted scope of these studies and the focus on one or two municipalities, some contradictory findings have emerged, raising the question as to the generalizability of the findings.

The National Survey of Lead and Allergens in Housing (NSLAH) provided the opportunity to investigate the predictors of endotoxin contamination in housing in a nationwide sample designed to represent the U.S. population. For this study, we sampled five locations within each home and assessed a host of characteristics of the homes and occupants, yielding a robust data set. Prior reports from this survey explored the relationships between allergen and endotoxin exposures and the prevalence of adverse health outcomes. Our goal in this study was to determine the factors related to increased levels of endotoxin in homes to guide future health studies and public health interventions designed to reduce exposures.

## Methods

### Study design

This study used samples that we collected for the NSLAH. The study design, sampling, and assay methods for endotoxin have been published (Vojta et al. 2002). The associations of endotoxin concentrations with allergy, asthma, and wheezing have also been published (Thorne et al. 2005). We carried out this study in 831 housing units representative of the nation’s 96 million homes that allow children. The parent study received institutional review board approval, and study subjects gave written informed consent before their participation.

### Exposure assessment

Two field staff visited each participating household and administered an extensive questionnaire, conducted a home inspection, and collected samples from five locations (bedroom floors, family room floors, beds, kitchen floors, and family room sofas). The questionnaire included information on age, type and conditions of the home, and demographics and health of the residents (Vojta et al. 2002). Dust was vacuum-sampled into an in-line filter using a standardized protocol and then sieved (425 μm), aliquoted into lots of 100 mg, and frozen at −80°C. Samples were then assayed for endotoxin and common allergens (Vojta et al. 2002). A 50-mg subsample of each dust sample was extracted with 1.0 mL pyrogen-free water containing 0.05% Tween-20 and analyzed for endotoxin using the kinetic chromogenic *Limulus* amebocyte lysate assay (Thorne 2000). In total, 2,512 endotoxin determinations were linked with complete housing data and were available for statistical analysis. We excluded 43 samples collected from basements from statistical analyses (because of limited power), leaving 2,469 endotoxin values from 790 households.

### Meteorologic data

We obtained meteorologic data for study locations specified by longitude and latitude (to three decimal degrees) from the Oregon Climate Service PRISM data explorer for monthly high-resolution precipitation and temperature climate data (Oregon Climate Service 2008). Annual precipitation and annual maximum and minimum temperatures were obtained for the years in which samples were collected and applied each as indicators of local climatic conditions in the regression modeling as prediction variables.

### Statistical analysis

We performed bivariate analyses and repeated-measures analyses of variance (rANOVAs) to assess the relationship between each housing or occupant characteristic and the level of endotoxin concentration [endotoxin units (EU) per milligram] and endotoxin load (EU per square meter). Endotoxin was evaluated as a continuous variable with logarithmic transformation. In the bivariate analyses, endotoxin levels were summarized using geometric means (GMs) and comparisons were made using ANOVAs.

For the rANOVA, we preliminarily identified 37 possible predictors of log-transformed endotoxin concentrations or loads measured at five different locations for each household, based on knowledge gleaned through previous research and the bivariate analysis results. Set 1 consisted of demographic factors, set 2 consisted of characteristics of the home, set 3 included questionnaire data on pets and vermin, set 4 included field-staff–observed evidence of household characteristics, and set 5 consisted of factors specific to bedrooms. We determined the optimal subset of these predictors using an rANOVA-based model selection process, with sampling locations treated as repeated measures and each household treated as an individual observation. In effect, the rANOVA approach characterizes relationships between predictors and the distribution of multiple related endotoxin measurements in a household.

Estimation and rANOVA model optimization were based on a maximum-likelihood procedure using the Akaike information criterion (AIC) statistic. We implemented a hierarchical model selection procedure in which we partitioned predictor variables of interest into five logical sets and sequentially selected the best subset of predictor variables from each set using an exhaustive search. We repeated the process using all possible orderings of the variable sets to obtain the optimal set of predictors. The best subset of bedroom-specific predictors was obtained by fitting models using only bedroom floor and bedroom bed endotoxin levels. Further details are described in Supplemental Material (available online at http://www.ehponline.org/members/2008/11759/suppl.pdf).

We applied sample weights in all analyses to account for housing unit selection probabilities, nonresponse, and poststratification. Taylor series linearization methods were used to obtain variance estimates adjusted for clustering associated with the multistage complex survey design, with the exception of the AIC-based rANOVA. Statistical analyses were conducted in SAS-callable SUDAAN (version 9.0; Research Triangle Institute, Research Triangle Park, NC) and SAS (version 9.1; SAS Institute Inc., Cary, NC).

## Results

**Figure 1:**
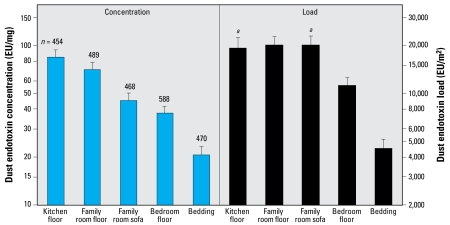
Endotoxin concentration (left) and endotoxin load (right) in the dust samples shown as GM and 95% confidence limits (error bars). We adjusted values for survey design information and sample weighting. ***a***Endotoxin load as EU per sample rather than EU per square meter.

This study is the first to evaluate domestic endotoxin levels over a wide geographic region and across demographic groups representing urban, suburban, rural; wealthy and poor; African American (black), Hispanic, and white; apartment dwellers and people living in multifamily or single family homes; children and adults; with or without pets; with and without allergy or asthma. This allowed us to develop an understanding of the predictors of domestic endotoxin for the entire United States. Figure 1 shows the GM concentrations of 2,469 surface samples collected from the kitchen floor, family room floor, family room sofa, bedroom floor, and bedding. Endotoxin concentrations in samples from the kitchen and family room floors were about 4-fold higher than concentrations in the bedding, and family room sofa and bedroom floor concentrations were approximately twice those in the bedding. Endotoxin load values demonstrated that bedroom floors were substantially less contaminated than family room floors, sofas, and kitchens but more than twice as contaminated as bedding. Although family room floors and sofas had lower endotoxin concentration than kitchen floors, the amount of dust was higher, so the endotoxin loads were comparable.

**Figure 2:**
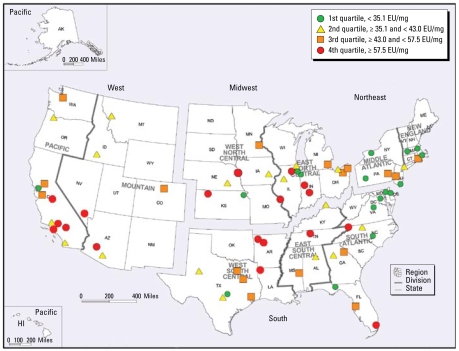
U.S. map showing the census regions, census divisions, and quartiles of the GM endotoxin concentration for all five sampling locations within homes, aggregated by PSUs of the survey.

Tables 1–3 show potential predictors of endotoxin concentrations assessed in this study for bedroom floor, family room floor, and bedding samples. Table 1 lists household factors and their endotoxin concentrations (GM and *p*-values) compared with the referent subpopulation (the referent is the sub-population with no *p*-value listed). A number of household factors showed consistency as predictors of endotoxin across sampling locations. The West census region (illustrated in Figure 2) had higher endotoxin levels than the Northeast, South, or Midwest regions. When we analyzed this further using the nine U.S. census divisions, we found that the Pacific division (California, Oregon, Washington) was the highest for all sampling locations and New England (Connecticut, Massachusetts, Maine, New Hampshire, Rhode Island, Vermont) was the lowest. The Pacific division spans 2,000 km from north to south and represents both warm, dry (e.g., San Diego, CA) and cool, wet climates (e.g., Portland, OR). In Figure 2 we have plotted quartiles of the GM endotoxin concentrations for all households and all household sampling sites within geographic primary sampling units (PSUs) [i.e., metropolitan statistical areas (MSAs) or rural counties]. On this map, for example, the orange square over Boulder County, Colorado, represents the unadjusted GM of 52 samples collected in the cities of Boulder and Longmont (population, 225,339; PSU weight, 20.357). The red circle in western Kansas represents 81 samples collected in five adjoining counties (combined population, 23,293; PSU weight, 91.333). Figure 2 illustrates that the high endotoxin values for the Pacific census division were primarily in Southern California. The New England and Middle Atlantic divisions plus Delaware, Maryland, Virginia, and the District of Columbia had no PSUs in the highest quartile and had 71% in the lowest quartile.

Another household factor relating to endotoxin was living in poverty, for which mean bedroom floor and bedding endotoxin levels were 56% (*p* = 0.003) and 58% (*p* = 0.021) higher than in nonimpoverished households, respectively. Households occupying two- or three-story homes including a basement (if present) had significantly lower bedroom floor (*p* = 0.002) and family room floor (*p* = 0.006) endotoxin. Homes on a single level or in multilevel apartment buildings had higher endotoxin. Having air conditioning, a stove exhaust fan, or an air filtration system were not significant predictors. Having electric heat as the main heating source was associated with higher bedroom (*p* = 0.012) and family room floor (*p* = 0.009) endotoxin than the other/none category. Also, whether the occupants lived in a single or multifamily dwelling or owned their home was not related to endotoxin in the homes.

Metropolitan status demonstrated higher values for MSAs with populations of > 1 million than for those with < 1 million that were significant for bed endotoxin (*p* = 0.035) and showed a trend for bedroom floor (*p* = 0.073) and kitchen floor (*p* = 0.080). Homes built before 1978 had higher endotoxin levels in family room floors (*p* = 0.040) but not in other locations.

Table 2 shows the GM and *p*-values for a variety of endotoxin source factors in domestic environments for bedding, bedroom floor, and family room floor endotoxin. Increasing numbers of people living in the household showed a very strong relationship with increasing endotoxin concentration, as did having children residing in the home. For family room floor endotoxin, the GM was 42.7 EU/mg for households with a single resident, 58.1 for two-member households (*p* = 0.019), between 76.8 and 79.0 for three or four residents (*p* < 0.005), and 87.0 for households with > four residents (*p* < 0.001). We also observed this trend for bedroom floor and bedding endotoxin but it was less dramatic. Having a child or children in the home was significantly associated with higher endotoxin for bedroom floors (*p* < 0.001), family room floors (*p* = 0.028), and bedding (*p*< 0.001).

Several other potential source factors were significantly associated with bedroom floor endotoxin. Current pets or pets in the household in the past 6 months and current or past dogs or cats were significant (all *p* ≤ 0.001; Table 2). Also significant were cockroach problems in the past year (*p* = 0.026) and, for family room floors, cigarette smoking (*p* = 0.004). We found no effect on endotoxin of dehumidifier use or season in which we sampled the household.

During household visits, our field staff conducted a walk-through survey noting specific factors relating to characteristics of the home. Table 3 lists staff-observed factors and their relationship with endotoxin concentrations. For both bedroom floors and family room floors, evidence of smoking (*p* = 0.012; *p* < 0.001), cockroach stains (*p* = 0.041; *p* = 0.009), and food debris (*p* = 0.044; *p* < 0.001) were significant predictors of endotoxin. Observed mold or mildew in the room was associated with higher bedroom endotoxin but was rarely observed (21 of 581). Carpeted floor, room air conditioner, and room air cleaning device were not significant predictors. Extreme room temperatures on the day of the survey [i.e., < 18°C (65°F) or > 29°C (84°F)] were associated with higher endotoxin concentration for bedroom floors (*p* = 0.008) and family room floors (*p* = 0.033). Relative humidity in the room on the survey day was not a factor for family room floor or bedding endotoxin. However, for bedroom floor endotoxin, relative humidity < 40% was associated with higher endotoxin than the other four humidity ranges from 40% to > 69%. Field staff recorded whether or not the bed in the sampled bedroom was equipped with an impermeable cover for the mattress, box spring, or pillow. Interestingly, all three covers were significantly associated with higher bedroom floor endotoxin concentration (Table 3). Having a stuffed animal (e.g., teddy bear) in the bed also increased bedding endotoxin (*p*= 0.024).

Table 4 lists data for significant predictors of kitchen floor endotoxin, which show that the kitchen floor had a distinct profile of endotoxin predictors. As with the other household sampling locations, kitchen endotoxin levels were significantly lower for the Northeast census region and the New England census division. Kitchen endotoxin was higher for those living in poverty (130 vs. 75 EU/mg; *p* = 0.001), with lower household income (*p* = 0.001), and with lower educational attainment (*p* = 0.021). Problems with cockroaches, live or dead cockroaches in the kitchen, and cockroach stains were all strong predictors of endotoxin levels (*p* < 0.001). Households reporting problems with cockroaches in the past 12 months had 2-fold higher endotoxin than did those without cockroaches. Within the subpopulation of those with cockroach problems, households where the residents sighted > 50 cockroaches per day (*n* = 7) had a mean kitchen floor endotoxin level of 838 EU/mg, 10-fold higher than the overall mean of 80.5 EU/mg. In addition, evidence of rodents (*p* = 0.002), cigarette smoking (*p* < 0.001), and mold or mildew (*p* = 0.02) were highly significant predictors of increased kitchen endotoxin concentration. In contrast to other locations in the homes, people of black race had significantly higher endotoxin in kitchen floor dust samples than did whites or other races (*p*= 0.005).

Next we sought to identify the optimal set of candidate predictors of household endotoxin using rANOVA with household as subject and the five sampling locations as repeated measures. To streamline the analysis, 37 candidate predictor variables were partitioned into five logical sets (S1–S5) shown in Table 5. After all permutations were explored, the model shown in Table 6 yielded high predictive values with strong statistical significance. Coefficients for sampling locations mirror the data shown in Figure 1, with bedding lowest and kitchen floor highest in endotoxin concentration. With the New England census division as the referent, Mountain, West North Central, and Pacific were 73–91% higher (*p* < 0.001) in household endotoxin concentration. Higher endotoxin concentration was associated with lower educational attainment (*p* = 0.014), children in the home (*p* = 0.035), currently having a dog in the household (*p* < 0.0001), problems with cockroaches in the past 12 months (*p* = 0.0022), field-staff–observed food debris (*p* = 0.029), cockroach stains (*p* < 0.0001), and evidence of smoking (*p* = 0.0087). When we ran the analysis for bedroom bedding and included floor endotoxin alone and S5 variables, the only additional variable from S5 that emerged was having an encapsulating mattress case on the sampled bed (*p* = 0.048). The rANOVA analysis for endotoxin load (Table 6) revealed that sampling location, census division, education, dog in home, problems with cockroaches, food debris, and cigarette smoking were significant predictors (*p* < 0.0001 for all). Additional predictors for endotoxin load were cat in home (*p* = 0.0035), mold/mildew observed (*p* = 0.0012), and lower relative humidity (*p* < 0.0001). The rank ordering of endotoxin load by census division was somewhat different than for endotoxin concentration, although Mountain, West North Central, and Pacific were the highest for both measures of endotoxin and New England was the lowest or second lowest.

The finding of a geographic trend for higher endotoxin and data suggesting an effect of poor indoor temperature control, low humidity, and type of heating led us to consider if the local temperature range or amount of precipitation during the study year were related to endotoxin concentration in homes. We reasoned that measurement of temperature and humidity on a single day could produce misclassification and be a poor measure of typical local climate or usual indoor conditions. Using spatial coordinates for each of the study households, we queried the Prism data explorer for annual precipitation and maximum–minimum temperatures for the year in which we sampled the home. Linear regression analysis of these factors with endotoxin concentration in each sampling location revealed no relationship of these factors for bedroom or family room floor endotoxin (Table 7). However, precipitation during the study year was a significant predictor of bedding endotoxin (*p* = 0.033). Temperature maxima and minima were related to kitchen floor endotoxin (*p* = 0.001 and *p* = 0.013, respectively) but showed no relation with endotoxin for other sampling locations.

## Discussion

NSLAH has provided valuable information on the levels of allergens and endotoxin in the U.S. housing stock and the relationships between exposures to these agents and disease (Arbes et al. 2003, 2004; Cohn et al. 2004, 2006; Elliott et al. 2007; Salo et al. 2005, 2006; Thorne et al. 2005). NSLAH characterized how exposures to indoor allergens vary in U.S. homes. *Alternaria*, cat, and dog allergens were most often detected and were the allergens found at elevated levels in most homes. Although each allergen appeared to have a distinct set of predictors, levels were strongly associated with regional, ethnic, and socio economic factors.

We previously reported from NSLAH that increasing concentration of endotoxin in homes was a risk factor for increased prevalence of diagnosed asthma, asthma symptoms in the past year, current use of asthma medications, and wheezing (Thorne et al. 2005). The joint effect of exposure to > 19.6 EU/mg bedroom floor and bedding endotoxin on recent symptomatic asthma yielded an adjusted odds ratio of 2.83 compared with exposures below this level. In our previous study, we also demonstrated that there was a relatively weak correlation between endotoxin values across sampling locations within homes, with correlation coefficients between 0.12 and 0.44, demonstrating the importance of sampling multiple locations within homes.

Several previous studies have investigated predictors of endotoxin in residences. Gehring et al. (2004) analyzed bedding dust endotoxin data from a birth cohort study of allergy [the ongoing birth cohort study Influences of Lifestyle-Related Factors on the Immune System and the Development of Allergies in Childhood (LISA)] conducted in Munich and Leipzig, Germany. In their study, 28% of the households were single-family homes, whereas 85% of U.S. households are single-family homes, reflecting the high degree of home ownership in the United States. Gehring et al. (2004) found that dog, but not cat, ownership was a significant predictor of endotoxin concentration. Endotoxin in bedding dust increased with increasing numbers of household occupants (< 4 vs. ≥ 4). Another study of endotoxin predictors was conducted in Erfurt and Hamburg, Germany (Bischof et al. 2002). This case–control study of adult asthma and allergy was conducted in 405 homes with samples collected from living room floors (95% with carpets). Predictors of higher endotoxin were old buildings, lower-story residence, longer occupancy, infrequent vacuum cleaning, dog and cat ownership, and mouse infestation. No seasonal effect was observed, and no association of endotoxin with indoor temperature or relative humidity was found.

In the LISA study, infants’ beds averaged 5.8 EU/mg endotoxin and mothers’ beds averaged 3.0 EU/mg, both much lower than the 18.7 EU/mg measured in beds in our study. Bischof et al. (2002) found mean endotoxin levels of 33.0 EU/mg, also considerably less than our value of 63.9 EU/mg for family room floors. Differences in sampling and analysis methodologies could potentially account for some of the increase in U.S. values over those in Germany. Endotoxin analyses for these studies were run somewhat differently than our methodology. Our dust samples were extracted using pyrogen-free water with 0.05% Tween-20, whereas their extraction was in pyrogen-free water alone. They ran duplicate assays at a single dilution, whereas we ran four 2-fold dilutions.

A third study analyzed data from living room carpets in 77 suburban homes in Wellington, New Zealand (Wickens et al. 2003). Important predictors of floor endotoxin concentration in the adjusted model were total occupants (2–4 vs. ≥ 5), maximum relative humidity (> 70.8% vs. < 70.8%), age of vacuum cleaner (older vs. newer than 1 year), and steam cleaning or shampooing the carpet. Factors not related to endotoxin concentration included having a cat, visible dampness or mold, and carpet type. That study was not able to assess differences in geography, housing type, poverty, or race.

Park et al. (2001) studied a cohort of children of parents with allergies or asthma living in the Boston area and evaluated factors associated with recurrent wheezing. Higher endotoxin concentration in family room floor dust was associated with having a dog, whereas being of black race was associated with significantly lower family room floor endotoxin. Family income was not a predictor of endotoxin in their multivariate analysis. Consistent with our study, their mixed-models analysis demonstrated that kitchen floors were higher and bedroom floors lower in endotoxin concentration compared with family room floors (Abraham et al. 2005). This is likely because bedrooms typically are not trafficked by all family members as are family rooms, whereas kitchens have more potential sources of endotoxin. In contrast to our nationwide study, Abraham et al. (2005) found that fall and winter sampling was associated with lower endotoxin. The lack of a seasonal effect in our study likely reflects the wide variation of climate in the United States. Although winter in Boston may produce dryer and colder indoor air, indoor winter conditions may be wetter (more rain) and warmer (air conditioning off) in U.S. population centers of the Southwest.

Consistent with these prior studies, we found that a higher number of occupants and dog ownership were important predictors of higher endotoxin. Age of the building was a significant factor, but only for family room endotoxin. In contrast to these studies, we found that geographic location, children in the home, poverty, cockroach infestation, smoking in the home, and, for some sampling locations, cat ownership were important factors. Several of these factors could not be investigated in the prior studies due to study design limitations (e.g., limited geography, single sampling location within homes, lack of diversity of population or home type, affluent population).

Gram-negative bacteria grow in ecologic niches that provide sufficient water, nutrients, oxygen, and heat. Dead or quiescent bacteria and cell-wall fragments composed of endotoxin can be transported in air or tracked in with dust and soil. Humans and pets harbor these organisms in the gut and on the skin, from which they are shed. Thus, larger families, children in the home, and dog ownership contribute to household endotoxin. Spoiling food and cockroach carcasses and feces are additional sources of endotoxin. Although cigarettes have a small amount of endotoxin, the association in this study with evidence of cigarette smoking is likely related to general home hygiene rather than dissemination of endotoxin through smoking. Lower educational attainment and living in poverty are predictors of endotoxin likely because of their association with poorer-quality housing, introduction of endotoxin via work clothes brought into the home, and a deficiency of home hygiene.

A significant strength of NSLAH is the characterization of predictors of endotoxin over a wide range of geography and population demographics in multiple locations within homes. The bivariate analyses (Tables 1–4) and the rANOVA (Tables 6 and 7) showed that the New England census division had the lowest levels of endotoxin for all five sampling locations in the homes and that the Pacific census division had the highest for four of the five. Nationwide, the highest combined endotoxin was measured in St. Louis, and the second and third highest were Los Angeles and Santa Clara counties in California. Figure 2 illustrates that New England, the Middle Atlantic, and the northern states of the South Atlantic census divisions had lower endotoxin. The southwestern United States, including California, Nevada, and Arizona, had higher levels. This is perhaps counterintuitive given the warm and often dry climate of this region. It is commonly assumed that because endotoxin arises from bacteria, and bacteria thrive in water, higher endotoxin will be associated with more humid climates. This has been found to be the case with molds and house dust mites. However, although typical indoor molds require water activities of only 0.8, bacteria require water activities of ≥ 0.97 and therefore grow on damp to wet substrates. Elevated humidity in the absence of wet surfaces or stagnant water in HVAC systems will not achieve water activity levels sufficient to provide an ecologic niche to support the growth of bacteria. Evaporative coolers, or swamp coolers, are a type of air conditioning found mostly in the Southwest that draws dry outside air through wetted pads to lower air temperature by evaporative cooling. This type of air conditioning was used in 14 of the households evaluated and was associated with significantly higher endotoxin in the bedding (*p* = 0.023) but was not significantly different for other sampling locations.

Main heating source and temperature control were important factors for family room and bedroom floor endotoxin. In bivariate analyses, having temperatures in the family room between 18°C and 23°C or in the bedroom between 24°C and 29°C was associated with lower endotoxin compared with more extreme temperatures (> 29°C or < 18°C). Electric heating was associated with higher endotoxin concentrations compared with the other/none category, and gas heating fell in between. Temperature control and heating systems vary regionally. Homes in areas with cold winters more often rely on gas heating, whereas homes in warmer climates may have only electric space heaters or no heating systems. We retained neither temperature in room nor heating source in the rANOVA models, likely due to their strong correlation with census division (chi-square test of independence for census division and heating source, *p*< 0.0001).

We performed the rANOVA in an attempt to determine which factors independently best predict endotoxin in the five sampling locations. The resulting model explained 30% and 52% of the variation in the log-transformed endotoxin concentration and load, respectively, beyond that explained by differences among the sampling locations themselves. This suggests the possibility of population sub-sampling and use of modeling to impute values for endotoxin. The rANOVA confirmed differences between sampling locations within homes and the distribution by census divisions. The rANOVA also demonstrated that lower educational attainment and presence of food debris and cockroaches are important predictors of endotoxin in homes. The additional factors of children and dogs in the household suggest that poor housing conditions and high occupancy are important factors leading to higher endotoxin exposures. Indeed, we tested other models and demonstrated that number of people in the household and living in poverty were important factors strongly correlated with children in the home and lower educational attainment, respectively. Pairwise tests of independence demonstrated strong covariance of lower educational attainment with both living in poverty and lower household income (chi-square test, *p* < 0.0001 for both).

Our study has several limitations regarding prediction of factors associated with endotoxin exposure. First, sampling was performed on a single day for each household. Thus, the dust sample and environmental data collected on that day were assumed to be representative of that household. Second, as is frequently done, we used measurements of reservoir dust endotoxin as a proxy for personal inhaled endotoxin exposure. Repeated measures of breathing zone endotoxin while subjects are awake and sleeping are difficult to obtain in a large study. Reservoir dust sampling likely reflects exposures sustained over a long period of time and has been shown to be associated with a variety of respiratory health outcomes (Thorne et al. 2005). In addition, it is likely a more stable estimate of exposure than a single-time-point air sample. Third, some of the data were based on interviews with the adult household resident. It is possible that responses to potentially sensitive questions such as indoor smoking or cockroach infestation were subject to reporting bias. However, this is partially mitigated by household observation data systematically reported by field staff. This study was strengthened by the fact that the weighted characteristics of the survey sample produced results indicative of the nation as a whole. The national scope of the study allowed us to investigate region and climate for their influence on indoor endotoxin concentrations.

## Conclusions

is nationwide study, representative of the U.S. housing stock, demonstrated that the concentration of endotoxin in house dust depends on the location sampled within the home and region of the country. Endotoxin concentrations increased with children or more occupants in the home, dogs present in the home, lower educational attainment, living in poverty, observed food debris, evidence of cockroach infestation, and evidence of cigarette smoking. The presence of stuffed animals in the bed and having an impermeable mattress cover were associated with higher bed endotoxin. In contrast to indoor molds and mite allergens, endotoxin was not associated with having air conditioning, dehumidifier use, or stove fans that exhaust outside. Neither race nor ethnicity emerged as independent predictors of household endotoxin. This study shows that the burden of domestic endotoxin exposure is disproportionately borne by families living with poor home hygiene. Public health interventions to reduce exposure to endotoxin should include improving housing conditions, eliminating cockroach infestations, reducing cigarette smoking indoors, and removing mold and mildew in homes. In addition to lowering endotoxin exposure, these interventions would reduce exposures to allergens and other asthma triggers.

## Figures and Tables

**Table 1:** Household predictors of endotoxin concentration in bedroom floors, family room floors, and bedding.

		Bedroom floor			Family room floor			Bedding		
Predictor	Subpopulation	No.	GM (EU/mg)	*p*-Value^a^	No.	GM (EU/mg)	*p*-Value^a^	No.	GM (EU/mg)	*p*-Value^a^
Census region	Northeast	96	29.1		72	51.4		82	16.4	
	South	210	33.6	0.407	158	62.0	0.402	161	16.9	0.885
	Midwest	137	37.4	0.174	139	67.6	0.152	114	18.9	0.542
	West	145	44.3	0.035*	120	75.6	0.068	113	25.0	0.046*
Census division	New England	30	24.7		21	31.1		29	13.7	
	South Atlantic	80	28.2	0.538	63	53.2	0.012*	61	15.3	0.750
	Middle Atlantic	66	33.4	0.175	51	75.8	0.000**	53	19.4	0.355
	West South Central	80	33.5	0.133	61	62.0	0.002**	56	19.8	0.252
	West North Central	60	35.7	0.078	59	64.5	0.000**	59	25.0	0.062
	East North Central	77	38.7	0.031*	80	69.9	0.000**	55	14.6	0.887
	East South Central	50	40.8	0.017*	34	74.3	0.042*	44	15.6	0.671
	Mountain	60	42.0	0.019*	43	67.2	0.000**	53	21.6	0.165
	Pacific	85	47.2	0.002**	77	83.4	0.000**	60	31.0	0.011*
Metro status	MSA < 1 million	302	32.6		249	61.2		228	16.6	
	Non-MSA	105	34.4	0.589	83	69.8	0.410	86	19.0	0.396
	MSA ≥ 1 million	181	42.1	0.073	157	64.3	0.744	156	22.6	0.035*
Housing unit type	Multifamily	88	27.2		75	61.2		71	16.3	
	Single family	500	36.8	0.103	414	64.3	0.786	399	19.1	0.447
Housing unit year category	1978 or newer	156	34.9		128	52.8		125	18.3	
	Older than 1978	432	35.5	0.910	361	69.9	0.040*	345	18.8	0.868
Race	Black	90	26.5		79	73.7		67	19.2	
	Other	54	30.5	0.519	40	81.2	0.719	43	19.2	1.000
	White	437	37.4	0.021*	363	61.8	0.260	351	18.8	0.915
Ethnicity	Non-Hispanic	520	34.9		443	63.0		414	18.0	
	Hispanic	62	39.5	0.567	42	73.1	0.517	51	27.2	0.095
Household income	≥ $30,000	327	31.8		266	63.4		255	18.2	
	< $30,000	235	41.2	0.045*	195	64.8	0.864	186	19.7	0.564
Living in poverty	No	450	32.9		378	62.6		355	17.6	
	Yes	106	51.5	0.003**	83	78.4	0.171	81	27.8	0.021*
Own or rent home	Rent	209	34.0		172	63.1		172	20.2	
	Own	377	35.9	0.648	315	64.1	0.914	296	17.9	0.360
Education after high school	Some	398	31.9		326	61.9		307	17.4	
	None	190	44.5	0.005**	163	68.6	0.357	163	21.6	0.183
No. of stories, including basement	2–3	307	30.7	0.002**	251	54.8	0.006**	262	17.4	0.164
	≥ 4	36	34.2	0.353	40	75.1	0.929	32	16.5	0.346
	1	243	42.8		196	76.5		174	21.4	
Main heating source	Other/none	111	28.5		89	50.9		93	18.2	
	Gas	302	35.8	0.058	252	63.2	0.174	247	19.8	0.543
	Electric	173	40.4	0.012*	146	78.1	0.009**	129	17.1	0.737
Air conditioning in home	Yes	463	35.0		378	64.5		368	17.6	
	No	124	36.1	0.737	110	61.3	0.722	101	22.9	0.105
Fan that exhausts stove to outside	No	133	30.8		110	62.8		118	17.7	
	Yes	128	34.7	0.445	114	71.8	0.418	92	24.4	0.084
Air filtration system in home	No	502	34.8		421	63.9		399	18.7	
	Yes	73	37.6	0.621	55	57.7	0.517	60	17.7	0.807

MSA, metropolitan staitistical area.

a Based on *t*-statistics using log-transformed endotoxin concentration.

* *p* < 0.05.

** *p* < 0.01.

**Table 2:** Endotoxin source as predictors of endotoxin concentration in bedroom floors, family room floors, and bedding.

		Bedroom floor			Family room floor			Bedding		
Endotoxin source	Subpopulation	No	GM (EU/mg)	*p*-Value	No.	GM (EU/mg)	*p*-Value	No.	GM (EU/mg)	*p*-Value^a^
No. of people living in the home	1	90	30.7		84	42.7		72	16.7	
	2	183	28.5	0.668	145	58.1	0.019*	152	13.3	0.296
	3	119	37.7	0.316	97	79.0	0.004**	85	23.6	0.067
	4	113	47.0	0.103	98	76.8	0.000**	93	25.5	0.073
	> 4	83	50.0	0.012*	65	87.0	0.000**	68	32.8	0.003**
Children < 6 years of age living in the home	No	465	33.1		397	62.3		377	16.4	
	Yes	121	49.3	0.001**	91	74.4	0.363	90	38.5	0.000**
Children < 18 years of age living in the home	No	313	29.6		267	57.8		246	14.3	
	Yes	274	47.0	0.000**	221	75.8	0.028*	222	28.7	0.000**
Pets in home in the last 6 months	No	258	27.2		221	63.5		220	16.2	
	Yes	328	43.0	0.000**	267	64.1	0.927	249	21.2	0.083
Pets currently in the home	No	286	27.7		242	60.9		244	15.8	
	Yes	299	44.3	0.000**	245	66.7	0.401	223	22.5	0.019*
Dogs in home in the last 6 months	No	365	29.4		304	62.0		308	17.3	
	Yes	218	46.2	0.000**	181	66.9	0.561	159	21.4	0.221
Dogs currently in the home	No	391	30.8		327	59.5		329	17.6	
	Yes	194	46.3	0.001**	160	73.2	0.051	138	21.3	0.209
Cats in home in the last 6 months	No	426	31.7		364	64.8		349	16.5	
	Yes	157	45.8	0.001**	121	61.4	0.679	119	26.1	0.012*
Cats currently in the home	No	443	31.9		376	63.8		358	16.6	
	Yes	142	47.9	0.000**	111	64.0	0.974	109	27.2	0.010*
Season home was sampled	Summer	184	32.2		161	71.3		156	16.7	
	Fall	268	34.3	0.614	231	62.8	0.443	206	18.8	0.528
	Winter	136	41.7	0.069	97	56.1	0.178	108	21.5	0.134
Problems with cockroaches in the past 12 months	No	461	33.0		375	60.1		372	17.8	
	Yes	126	49.4	0.026*	113	82.1	0.046*	97	23.5	0.051
No. of cockroaches seen per day on average	< 5	69	44.8		61	83.7		53	25.2	
	5–50	17	111.6	0.016*	15	111.3	0.328	15	23.9	0.907
	> 50	7	62.3	0.211	6	175.3	0.222	7	39.8	0.373
Cockroaches controlled by an exterminator	Yes	35	40.5		29	81.2		28	23.6	
	No	90	53.7	0.262	83	81.4	0.994	68	23.9	0.957
Any insecticides, bug sprays, or roach motels used	No	23	43.7		23	92.3		18	18.2	
	Yes	102	50.4	0.546	90	79.7	0.537	79	24.8	0.432
Cigarette smokers in household	No	340	32.7		283	56.1		268	17.2	
	Yes	245	39.1	0.119	204	76.7	0.004**	200	20.9	0.070
Frequency of cigarettes smoked inside per day	Never	51	26.8		39	88.7		40	18.4	
	< Once	15	35.9	0.570	11	59.7	0.145	10	26.9	0.445
	1–3 times	21	34.7	0.430	18	62.9	0.277	16	18.3	0.990
	4–10 times	55	33.7	0.319	41	52.6	0.079	51	21.1	0.643
	> 10 times	97	53.5	0.001**	89	92.4	0.850	76	21.9	0.489
Cigar, pipe, etc., smokers in household	No	537	35.8		441	63.4		429	18.4	
	Yes	48	29.7	0.251	45	64.2	0.946	38	21.9	0.524
Use of dehumidifier in the home	Yes	85	33.9		69	67.3		79	16.3	
	No	492	35.8	0.682	412	62.9	0.676	386	19.1	0.306
Last time floor or carpet was cleaned	≥ 1 week ago	278	31.3		208	61.0		203	16.8	
	< 1 week ago	274	39.7	0.020*	270	65.9	0.562	235	20.9	0.183

a Based on *t*-statistics using log-transformed endotoxin concentration.

* *p* < 0.05.

** *p* < 0.01.

**Table 3:** Field-staff–observed predictors of endotoxin concentration in bedroom floors, family room floors, and bedding.

		Bedroom floor			Family room floor			Bedding		
Predictor	Subpopulation	No.	GM (EU/mg)	*p*-Value^a^	No.	GM (EU/mg)	*p*-Value^a^	No.	GM (EU/mg)	*p*-Value^a^
Evidence of smoking in the room	No	504	32.6		374	58.1		407	18.7	
	Yes	77	55.9	0.012*	109	88.7	0.001**	59	17.5	0.575
Cockroach stains in the room	No	566	34.1		471	62.6		454	18.4	
	Yes	12	70.6	0.041*	11	142.2	0.009**	11	29.4	0.201
Live/dead cockroaches in the room	No	570	34.4		465	63.3		458	18.4	
	Yes	10	65.4	0.122	18	78.5	0.367	8	31.1	0.263
Evidence of rodents in the room	No	566	35.2		476	63.4		456	18.4	
	Yes	13	22.7	0.567	6	106.9	0.179	10	30.0	0.173
Food debris in the room	No	495	33.2		386	57.7		404	18.0	
	Yes	85	50.1	0.044*	97	95.5	0.000**	61	23.2	0.126
Mold/mildew observed in the room	No	560	34.3		461	62.9		446	18.3	
	Yes	21	61.9	0.058	22	93.2	0.151	20	25.6	0.048*
Other moisture evidence in the room	No	542	34.4		457	62.6		430	18.3	
	Yes	39	45.8	0.174	26	95.9	0.109	36	22.2	0.203
Floor surface carpeted	No	75	35.4		60	57.6		88	18.4	
	Yes	490	34.7	0.883	415	65.0	0.500	364	18.5	0.969
Temperature in room (°C)	< 18	27	57.6		28	85.9		20	21.1	
	18–23	233	37.7	0.068	202	56.4	0.033*	186	19.5	0.746
	24–29	278	30.4	0.008**	215	66.9	0.275	223	17.2	0.385
	> 29	39	50.6	0.656	38	79.0	0.744	31	20.9	0.982
Relative humidity in room (%)	< 40	116	46.9		105	64.9		88	19.9	
	40–49	188	34.5	0.030*	140	60.3	0.666	156	17.6	0.449
	50–59	128	31.3	0.016*	123	74.8	0.401	106	19.8	0.992
	60–69	98	31.0	0.008**	74	50.5	0.260	84	16.8	0.467
	> 69	49	30.6	0.014*	44	77.5	0.472	28	18.5	0.785
Room air conditioner	No	521	34.6		400	62.7		423	18.5	
	Yes	56	34.9	0.964	83	69.9	0.474	42	18.9	0.920
Room air cleaning device	Yes	7	24.3		11	75.6		7	13.6	
	No	570	34.8	0.127	471	63.5	0.132	458	18.7	0.441
Mattress cover on bed	No	417	31.1					370	17.4	
	Yes	143	46.0	0.001**				85	26.9	0.051
Box spring cover on bed	No	452	32.7					392	17.7	
	Yes	109	44.0	0.036*				65	26.6	0.082
Pillow cover on bed	No	433	32.2					377	17.6	
	Yes	128	44.0	0.018*				80	25.4	0.111
Stuffed animal(s) in bed	No	431	34.1					357	17.3	
	Yes	130	35.0	0.822				101	23.8	0.024*

a Based on *t*-statistics using log-transformed endotoxin concentration.

* *p* < 0.05.

** *p* < 0.01.

**Table 4:** Predictors of endotoxin concentration in kitchen floors.

		Kitchen floor		
Predictor	Subpopulation	No.	GM (EU/mg)	*p*-Value^a^
Census region	Northeast	86	54.3	
	West	111	81.3	0.024*
	Midwest	106	89.0	0.005**
	South	151	94.4	0.004**
Census division	New England	28	43.5	
	Middle Atlantic	58	65.9	0.022*
	East North Central	55	76.2	0.003**
	Mountain	40	77.2	0.000**
	East South Central	38	81.5	0.162
	Pacific	71	85.7	0.018*
	South Atlantic	52	92.0	0.005**
	West South Central	61	104.6	0.000**
	West North Central	51	107.0	0.000**
Metro status	MSA < 1 million	218	69.2	
	Non-MSA	92	89.8	0.102
	MSA ≥ 1 million	144	93.1	0.080
Housing unit type	Single family	393	75.9	
	Multifamily	61	126.1	0.011*
Race	White	343	75.8	
	Other	37	76.6	0.957
	Black	68	118.3	0.005**
Household income ($)	≥ 30,000	260	66.1	
	< 30,000	171	114.8	0.001**
Living in poverty	No	354	75.2	
	Yes	75	130.0	0.001**
Own or rent home	Own	305	72.4	
	Rent	146	104.9	0.017*
Education after high school	Some	313	73.4	
	None	141	100.0	0.021*
No.of stories, including basement	2–3	238	72.8	0.064
	≥ 4	42	84.9	0.708
	1	173	92.9	
Main heating source	Other/none	102	70.2	
	Gas	237	77.0	0.557
	Electric	113	101.5	0.067
Cats in home in the last 6 months	Yes	119	66.9	
	No	331	85.9	0.062
Problems with cockroaches in the past 12 months	No	356	70.4	
	Yes	98	144.4	0.000**
No. of cockroaches seen per day on average	< 5	54	136.4	
	5–50	13	140.1	0.939
	> 50	7	838.4	0.000**
Cigarette smokers in household	No	265	68.9	
	Yes	187	101.1	0.007**
Evidence of smoking in the room	No	348	70.3	
	Yes	105	123.4	0.000**
Cockroach stains in the room	No	398	73.8	
	Yes	52	170.7	0.000**
Live/dead cockroaches in the room	No	413	74.9	
	Yes	39	204.7	0.000**
Evidence of rodents in the room	No	430	77.8	
	Yes	23	152.4	0.002**
Mold/mildew observed in the room	No	379	76.9	
	Yes	74	103.5	0.020*
Floor surface carpeted	No	364	73.8	
	Yes	76	100.8	0.068

a Based on *t*-statistics using log-transformed endotoxin concentration. Only predictors with *p*-values ≤ 0.10 are shown.

* *p* < 0.05.

** *p* < 0.01.

**Table 5:** Variables entered into the repeated measures ANOVA.

Set	Variable
S1	Census division (nine levels)
	Metro status (certainty MSA, MSA, non-MSA)
	Own or rent home
	Household income < $30,000/year
	Living in poverty
	Race (white, black, other)
	Education after high school (some, none)
S2	Housing unit type (single family, multifamily)
	Housing unit age (1978 or newer, older than 1978)
	No. of stories, including basement
	Main heating source (gas, electric, other/none)
	Air conditioning in home
	Fan that exhausts stove to outside
	Air filtration device in home
	Water or dampness in home in past 12 months
	Home often have mildewy or musty odor
	Dehumidifier used in home
	No. of people living in the home
	Household has children < 18 years of age
S3	Pets currently in the home
	Dogs currently in the home
	Cats currently in the home
	Problems with cockroaches in past 12 months
	Cigarette smokers in the home
S4	Carpet in room
	Temperature in room
	Relative humidity in room
	Mold/mildew observed
	Food debris observed
	Evidence of smoking
	Cockroach stains observed
	Live/dead cockroaches observed
	Evidence of rodents
S5	Encapsulating mattress case observed
	Encapsulating box spring case observed
	Encapsulating pillow case observed
	Stuffed animals in bed

**Table 6:** Major predictors of endotoxin concentration and endotoxin load from rANOVAs.

		Endotoxin concentration				Endotoxin load			
Predictor	Category	*p*-Value^a^	Estimate^b^	SE	*e*^β^	*p*-Value^a^	Estimate^b^	SE	*e*^β^
Sampling location	Bedding	< 0.0001	2.40	0.12	11.1	< 0.0001	0.61	0.076	1.84
	Bedroom floor		3.00	0.12	20.1		1.00	0.075	2.72
	Family room sofa		3.23	0.12	25.4		1.05	0.077	2.86
	Family room floor		3.56	0.12	35.3		1.21	0.076	3.35
	Kitchen floor		3.73	0.12	41.8		0.92	0.079	2.50
Census division	New England	< 0.0001	0.00			< 0.0001	0.007	0.065	1.01
	East South Central		0.22	0.13	1.25		0.072	0.065	1.08
	South Atlantic		0.28	0.13	1.32		0.033	0.063	1.03
	West South Central		0.39	0.12	1.48		0.077	0.059	1.08
	Middle Atlantic		0.45	0.13	1.57		0.000		
	East North Central		0.46	0.12	1.58		0.039	0.058	1.04
	Mountain		0.55	0.14	1.73		0.126	0.065	1.13
	West North Central		0.65	0.13	1.91		0.301	0.063	1.35
	Pacific		0.65	0.13	1.91		0.124	0.063	1.13
Education	None after high school	0.014	0.00			< 0.0001	0.00		
	Some after high school		−0.16	0.06	0.85		−0.14	0.032	0.87
Dog currently in the home	No	< 0.0001	0.00			< 0.0001	0.00		
	Yes		0.28	0.06	1.33		0.16	0.031	1.17
Problems with cockroaches in the past 12 months	No	0.0022	0.00			< 0.0001	0.00		
	Yes		0.26	0.08	1.29		0.18	0.041	1.19
Food debris observed	No	0.029	0.00			< 0.0001	0.00		
	Yes		0.15	0.07	1.16		0.17	0.035	1.19
Cockroach stains observed	No	< 0.0001	0.00			0.0027	0.00		
	Yes		0.60	0.14	1.81		0.24	0.081	1.28
Evidence of smoking									
Cigarette smokers in the home	No	0.0087	0.00			0.0007	0.00		
	Yes		0.19	0.07	1.21		0.10	0.030	1.11
Household has children < 18 years of age	No	0.035	0.00			NS			
	Yes		0.13	0.06	1.14				
Housing unit year category	Older than 1978	NS				< 0.0001	0.00		
	1978 or newer						−0.13	0.032	0.88
Cat currently in the home	No	NS				0.0035	0.00		
	Yes						0.10	0.034	1.11
Mold/mildew observed	No	NS				0.0012	0.00		
	Yes						0.20	0.063	1.23
Relative humidity in home (%)	< 40	NS				< 0.0001	0.234	0.062	1.26
	40–49						0.043	0.058	1.04
	50–59						0.115	0.058	1.12
	60–69						0.002	0.060	1.00
	> 70						0.000		

NS, not significant (α = 0.05).

a Based on F-statistics for type-3 tests of overall significance of each factor.

b Coefficient estimates for the sampling locations represent the mean log-transformed endotoxin concentration (EU/mg) at each location, at the reference level of all other factors in the model. Coefficients for other factors represent the estimated additional effect associated with the indicated level of each factor.

**Table 7:** Consideration of potential role of local meteorologic data (*p*-values) during the study year on endotoxin concentration indoors.

Location	Maximum temperature (°C)	Minimum temperature (°C)	Precipitation (mm)
Bedroom floor	NS	NS	NS
Family room floor	NS	NS	NS
Bedding	NS	NS	0.033
Kitchen floor	0.001	0.013	NS
Family room sofa	NS	NS	0.081

NS, not significant (α = 0.05). We considered meteorologic factors separately to predict endotoxin concentration by location based on longitude and latitude of the household.
